# Fiber Visualization with LIC Maps Using Multidirectional Anisotropic Glyph Samples

**DOI:** 10.1155/2014/401819

**Published:** 2014-08-28

**Authors:** Mark Höller, Kay-M. Otto, Uwe Klose, Samuel Groeschel, Hans-H. Ehricke

**Affiliations:** ^1^Institute for Applied Computer Science (IACS), Stralsund University, Zur Schwedenschanze 15, 18435 Stralsund, Germany; ^2^MR Research Group, Department of Diagnostic and Interventional Neuroradiology, University Hospital Tübingen, Hoppe-Seyler-Straße 3, 72076 Tübingen, Germany; ^3^Department of Pediatric Neurology & Developmental Medicine and Experimental Pediatric Neuroimaging, University Children's Hospital, Hoppe-Seyler-Straße 1, 72076 Tübingen, Germany

## Abstract

Line integral convolution (LIC) is used as a texture-based technique in computer graphics for flow field visualization. In diffusion tensor imaging (DTI), LIC bridges the gap between local approaches, for example directionally encoded fractional anisotropy mapping and techniques analyzing global relationships between brain regions, such as streamline tracking. In this paper an advancement of a previously published multikernel LIC approach for high angular resolution diffusion imaging visualization is proposed: a novel sampling scheme is developed to generate anisotropic glyph samples that can be used as an input pattern to the LIC algorithm. Multicylindrical glyph samples, derived from fiber orientation distribution (FOD) functions, are used, which provide a method for anisotropic packing along integrated fiber lines controlled by a uniform random algorithm. This allows two- and three-dimensional LIC maps to be generated, depicting fiber structures with excellent contrast, even in regions of crossing and branching fibers. Furthermore, a color-coding model for the fused visualization of slices from T1 datasets together with directionally encoded LIC maps is proposed. The methodology is evaluated by a simulation study with a synthetic dataset, representing crossing and bending fibers. In addition, results from *in vivo* studies with a healthy volunteer and a brain tumor patient are presented to demonstrate the method's practicality.

## 1. Introduction

In diffusion-weighted magnetic resonance imaging (DW-MRI), only a few white matter visualization approaches have gained clinical relevance despite the introduction of several novel imaging techniques, such as high angular resolution diffusion imaging (HARDI). Among these, color-coded FA maps and tractography-based streamlines and stream tubes have had the widest clinical application. Color-coded FA maps combine the fractional anisotropy (FA) of each voxel with the color-coded principal eigenvector of the diffusion tensor [1]. Although from color-coded slice images the major pathways may be mentally reconstructed, they primarily reveal only local anisotropic information. In addition, the diffusion tensor model is only capable of resolving a single anisotropy direction for each voxel. In more complex fiber architectures, such as crossing, branching, and kissing fibers, diffusion tensor imaging is inadequate and may result in misleading and inaccurate interpretations [2, 3]. Geometric models using streamlines [4] and stream tubes [5] can be used to visualize results of deterministic and probabilistic tractography algorithms. Furthermore, more complex approaches using hyperstreamlines [6] and tensor lines [7] have also been explored. Whilst these methods reveal global relationships, such as connections between brain regions, they fail to reliably depict uncertainties in the presented fiber anatomy, due to problems of data acquisition and signal processing. Rather, they represent data interpretations and depend on processing parameters, including choice of seed regions, tracking algorithms, and track termination criteria. This is also true for results of feature extraction methods [8], which generate a pathway's complex hull, for example, by segmentation or fiber clustering [9–12].

In order to visualize the diffusion tensor's characteristics, such as anisotropic diffusion direction and magnitude, different types of glyphs are used. The first glyph to be applied to diffusion tensor imaging (DTI) data was an ellipsoid [13]. The shape of the diffusion ellipsoid depends on the local diffusion profile and is determined by three eigenvalues and corresponding eigenvectors. Other glyphs, constructed from geometric primitives like cuboids and cylinders, have also been proposed. Kindlmann [14] introduced superquadric glyphs, which combine symmetry properties of ellipsoids with the shape and orientation of cuboids and cylinders. This facilitates the distinction between different shapes of the local diffusion profile. For the visualization of HARDI datasets orientation distribution function (ODF) glyphs may be used. These are constructed by the deformation of a sphere surface according to ODF values distributed over a half sphere. Directional color-coding of ODF glyphs helps to reveal local anisotropy directions [15, 16]. Glyphs can give the full diffusion profile information for each voxel, whereas the geometry of pathways and thus connectivity information are difficult to identify. Merging ellipsoids [17] is a technique used to visualize diffusion tensor imaging data by combining local tensor information with global connectivity features. Ellipsoids are placed along integral curves, generated by tractography, and merged under the control of weighted influence functions, derived from tensor properties.

In visualization theory there is another class of algorithms, visualization by textures, which may help to bridge the gap between local and global representations of the diffusion field. Triggered by advances in computer graphics hardware, texture-based visualization has gained great attention in the scientific community and various algorithms have been proposed, some of which may be applied to flow fields. Among these, line integral convolution (LIC), which was originally proposed by Cabral and Leedom [18], seems to be applicable to DW-MRI datasets. This technique smoothes a noise input image with a vector field, using a convolution kernel, which is locally adapted by vector field integration. Mcgraw et al. applied the LIC algorithm to a smoothed field of principal eigenvectors to visualize rat spinal cords [19]. Hsu was one of the first to apply LIC to a diffusion tensor field, using the tensor's principal eigenvector to guide the construction of a fixed-length filter kernel [20]. When using LIC, the choice of the input noise texture strongly influences the resulting image. Banks and Kiu [21] applied a multifrequency input noise scheme to visualize flow magnitude and direction. By modifying different parameters of the input image, Hotz et al. [22] encoded the eigenvalues of a positive-definite metric with the same topological structure as the tensor field. Wunsche and Linden [23] proposed a three-dimensional LIC volume to be visualized by direct volume rendering. It is also possible to compute a single LIC image for each of the three eigenvectors and overlay the resulting images to get a fabric-like texture [22]. In a previous paper we described an adaptation of the LIC method to high angular resolution diffusion imaging (HARDI), utilizing orientation distribution functions (ODFs) as representations of the local diffusion profile. Thus we were able to consider more than one anisotropy direction and correctly visualize crossing and kissing fiber pathways [24]. Moreover, we proposed a color-coding scheme for the directional encoding of LIC slices, enhancing fiber continuity perception. In contrast to streamline tractography methods, the LIC approach is easily parameterized, it does not require that seed or goal regions be defined, and it applies tracking only in a very small region around a voxel, thus avoiding error propagation along longer integral curves. Therefore, it is less prone to visualizing nonexistent tracts or failing to visualize existing fiber pathways. However, in our previous work, the LIC map lacked contrast, which made visualization of smaller fiber bundles difficult. In this paper we introduce a methodology for generating anisotropic glyph samples as an input pattern for an LIC algorithm. The geometry of glyph samples is derived from fiber orientation distribution functions (FODs) and according to the local diffusion profile can represent multiple anisotropy directions. As a glyph sample we use the three-dimensional geometry of the FOD shape model, rasterized with a superresolution voxel grid. We also use the more simple shape of multiple cylinders which are constructed in line with anisotropy directions. Placement and packing are controlled by the combination of a stochastic approach with local fiber line integration. This allows LIC maps to be generated with improved contrast, in which the viewer may visually track fibers, even through regions of crossing, branching, or kissing fiber pathways.

## 2. Method

**Figure 1:**
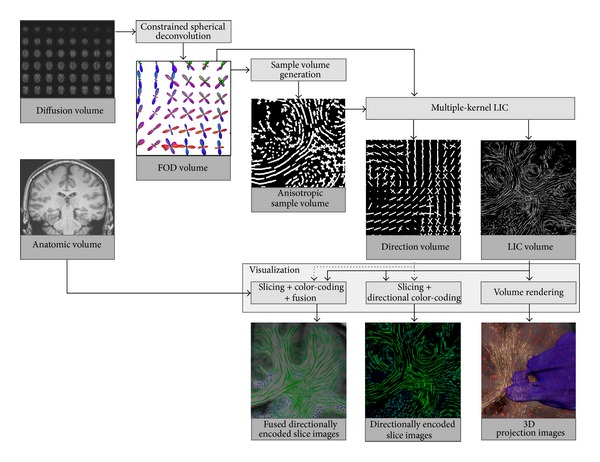
Overview of the most important steps and data elements of the overall method.

Originally, the LIC approach was designed for flow visualization by engraving a vector field's structure onto a noise texture. LIC is essentially a filtering technique that blurs an input texture locally along a given vector field, thus providing highly correlated voxel intensities along field lines. Initially, for each voxel of the input texture, a field line is integrated over a fixed number of voxels, using the voxel as a tracking seed. The field line is used as the kernel of a convolution operator, which averages voxel intensities along the line. In the method described here, a white noise texture is not used as originally proposed by Cabral and Leedom; rather an anisotropic spot pattern, which is continuously sampled along integral lines, is implemented. Furthermore, we propose a multikernel approach allowing more than a single anisotropy direction to be visualized for each voxel.  Figure 1 shows an overview of the approach adopted, depicting the most relevant processing steps and data elements. From the acquired HARDI diffusion dataset FODs are computed by spherical deconvolution. The FOD volume is used to create a high-resolution volume of glyph samples, which are used as the input pattern to a multiple-kernel LIC algorithm. The resulting LIC volume is a three-dimensional gray scale texture representing regional anisotropic behavior. Additionally, a direction volume is generated, in which the averaged anisotropy direction within the LIC convolution kernel is stored as a direction vector for each voxel. This is used for directional color encoding of slices through the LIC volume. In the visualization step, LIC slices can be fused with anatomic slice images, for example, from a T1 weighted dataset. By the application of volume rendering techniques, the LIC volume can be three-dimensionally visualized as a whole or after definition of a volume of interest. The details of (i) sample volume generation, (ii) multikernel LIC, and (iii) visualization are described below.

### 2.1. Generation of Anisotropic Glyph Samples

#### 2.1.1. FOD Glyphs and Cylindrical Glyphs

In order to prepare and structure input data for the LIC algorithm, various methods have previously been proposed. The most common approaches utilize high frequency white noise [18] or sparse noise [25, 26] fields. It has been demonstrated that, by applying dot-like structures, the contrast of the LIC output can be enhanced. Ellipses are structures well suited to this purpose, since they can represent local features of the flow field, such as flow direction and magnitude. As the shape of elliptical spots is defined by the parameters of the diffusion tensor, difficulties arise when trying to visualize multiple local fiber orientations. We have previously demonstrated that, by using ODF glyphs, more complex fiber anatomies can be visualized with the LIC method [24]. Several methods provide a higher angular resolution of kissing, branching, and crossing fibers in a voxel, including sharpening ODFs by regularization algorithms [27, 28], ODF reconstruction with constant solid angle (CSA) [29], tensor decomposition [30], or spherical deconvolution, leading to the fiber orientation distribution (FOD) function [31, 32]. Instead of elliptical or ODF-based samples, we propose the use of three-dimensional glyph samples derived from the FOD as the input pattern for the LIC algorithm. We decided to use the FOD method as it is highly relevant to clinical applications and FODs can easily be calculated from HARDI signals.

**Figure 2:**
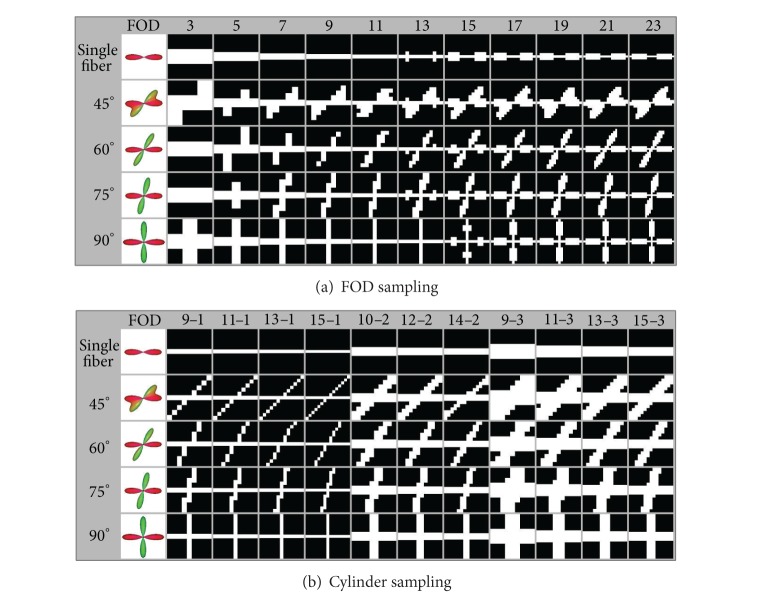
Glyph sampling with different anisotropy direction angles: (a) sampling of FOD glyph with sampling sizes from 3 × 3 × 3 to 23 × 23 × 23, (b) sampling of cylindrical glyphs with sampling sizes from 9 × 9 × 9 to 15 × 15 × 15 and cylinder width from 1 to 3.

In order to represent the shape of the FOD in a three-dimensional regular grid, we construct a superresolution LIC input grid with a spatial resolution in the order of 0.1 to 0.2 mm. Sampling of the FOD is performed by marking all voxels in the superresolution grid which are in contact with the FOD shape model, depicted in white and empty voxels in black (Figure 2(a)). The figure shows the results of different glyph sizes between 3 × 3 × 3 and 23 × 23 × 23 superresolution voxels. The figure clearly demonstrates that the accuracy of the representation improves with rising sampling ratio, which is particularly relevant to multidirectional FODs with smaller angles between fiber orientations. However, with an increasing sampling ratio we have to increase the size of the superresolution grid. This requires more computer memory and processing power. In evaluation experiments, we found sample sizes of 9 × 9 × 9 to 13 × 13 × 13 to be an acceptable compromise between accuracy of representation and computational workload. To place up to eight glyph samples within a voxel of the original diffusion dataset, a superresolution dataset with a spatial resolution of 1/18 to 1/26 of the voxel length of the original dataset is required.

**Figure 3:**
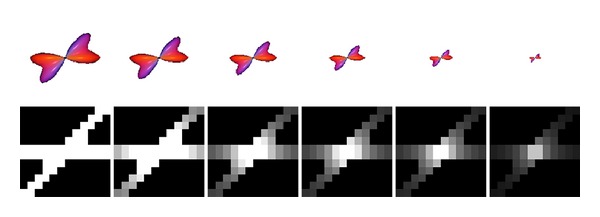
Gray value scaling of cylinders depending on the FOD amplitude.

Since the binary input pattern for the LIC algorithm should primarily represent fiber directions, it is sufficient to use the simpler glyph of a cylinder instead of the FOD. The main axis of the cylinder denotes a fiber direction, which is derived from a local FOD maximum. If an FOD has only one local maximum, a single cylinder is constructed with orientation and length defined by the FOD maximum. Multiple crossing cylinders represent an FOD with two or more local maxima, thus making use of multicylindrical samples. For each maximum we place one cylinder into the grid. Since the width of the cylinder is not determined by signal parameters or the FOD's shape, it can be freely chosen depending on the grid resolution. As an extreme, the cylinder can be represented by a straight line, which is sufficient, particularly when using low-resolution grids.  Figure 2(b) shows that for sample sizes of 9 to 13 the sampled cylinders do not differ too much from FODs, and fiber orientation angles of 45 degrees can be resolved. Additionally, the amplitude of the FOD is encoded using a gray scaling scheme. High amplitudes end up with cylinders in full white, whereas short amplitudes result in cylinders with gray scales from light gray to black. Thus, it is possible to regard local anisotropy information represented by the FOD's size (Figure 3).

#### 2.1.2. Superresolution Sampling

Distributing samples over a regular grid as an input to the LIC algorithm can produce undesirable effects, such as visual artifacts by line shifts and breaks. Feng et al. constructed a set of nonoverlapping ellipses as an input to a generalized anisotropic Lloyd relaxation process, resulting in textures similar to those generated by the reaction diffusion approach [33, 34]. Kindlmann and Westin proposed the distribution of glyphs over a nonregular grid. In this approach, potential energies between neighboring tensors were calculated and glyphs placed into a dense packing throughout the field. Glyph packing may be applied either to a slice or to the volume dataset as a whole [35]. Kindlmann et al. also used the tensor volume to simulate a reaction-diffusion process of two chemicals in an anisotropic medium in order to produce a texture of spots, distributed over a nonregular grid [36].

In the context of fiber visualization and diffusion signal processing, placement of samples for use with LIC should reflect the underlying fiber pattern as represented by the diffusion data. In order to avoid discontinuities in the fiber texture image produced by the LIC algorithm, it is useful to place samples along fiber pathways within the superresolution grid, so that samples are placed with improved fiber continuity, rather than on the regular grid of the original diffusion dataset. Therefore the following algorithm for sample placement is proposed.

1. Create a superresolution grid.
2. Randomly place seed points distributed over the superresolution grid by a uniform random technique.
3. To each seed point apply deterministic tracking over a short distance.
4. For each tracked streamline, place glyph samples (cylinders/FODs) along the streamline by tight packing.

Firstly, a superresolution grid is created. The resolution factor 1/*F*_*E*_ relates to the voxel length of the original diffusion dataset. Each voxel of the diffusion dataset is divided into *F*_*E*_ × *F*_*E*_ × *F*_*E*_ subvoxels. A diffusion dataset with 2.0 mm voxel size typically results in a superresolution dataset with a subvoxel size of approximately 0.1 mm. For the random placement of seed points, iterations are made until 1% of the points of the superresolution grid are filled with seeds. A rate of 1% was found to be sufficient to fill the grid with tightly packed glyph samples. The deterministic tracking uses interpolated FODs and integrates along local maxima, using a Runge-Kutta integration scheme. Tracking stops after *n* integration steps, where *n* is dependent on the grid's resolution and is chosen so that the tracking distance is in the order of the voxel size of the original diffusion dataset. This leads to streamlines with a maximum length of approximately 20 mm per direction. Along this short streamline up to 5 cylinders can be placed. The short tracking distance minimizes the cumulative tracking error ensuring that we do not follow nonexistent pathways.

A further stopping criterion is an FA threshold of 0.05 to avoid tracking out of the brain boundary. To avoid overlapping of glyph samples, samples are rejected if they intersect with any previously accepted sample.

**Figure 4:**
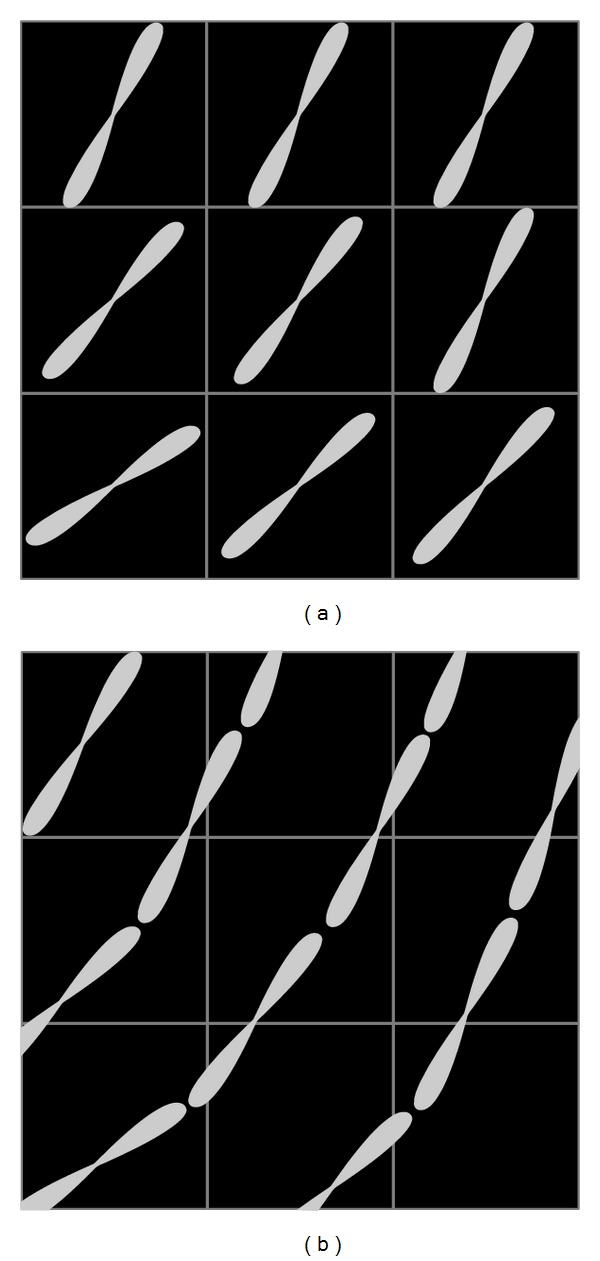
Comparison of input patterns using FOD glyphs: placement on regular grid (a) and continuous placement within superresolution grid (b).

**Figure 5:**
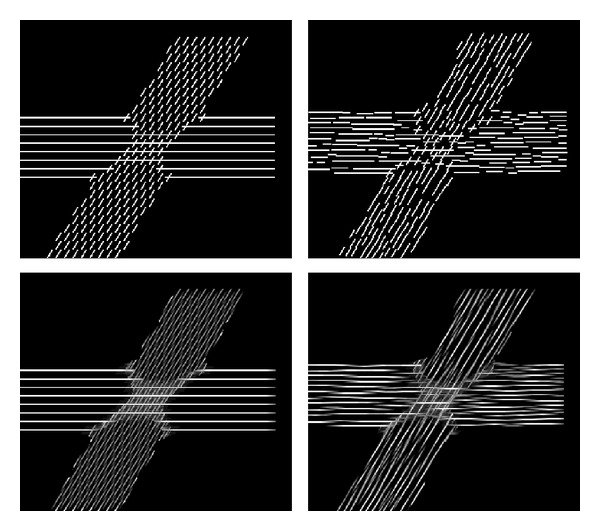
Comparison of cylindrical glyph samples (top) and LIC results (bottom) with sample placement on a regular grid (left) and continuous placement (right). The synthetic dataset contained two 5 mm thick fibers crossing at an angle of 60°. The cylindrical input patterns had a length of 12 subvoxels and a width of 2.

Figures 4 and 5 compare the sample placement strategies. In Figure 4, FOD glyphs were placed within the voxel raster of the original dataset (Figure 4(a)) and, alternatively, within the superresolution grid along tracked integration lines (Figure 4(b)).  Figure 5 compares the two placement strategies with cylindrical glyphs in crossing fibers. The upper row shows the LIC input patterns, whereas the corresponding LIC results are depicted in the lower row.

### 2.2. Multikernel LIC

**Figure 6:**
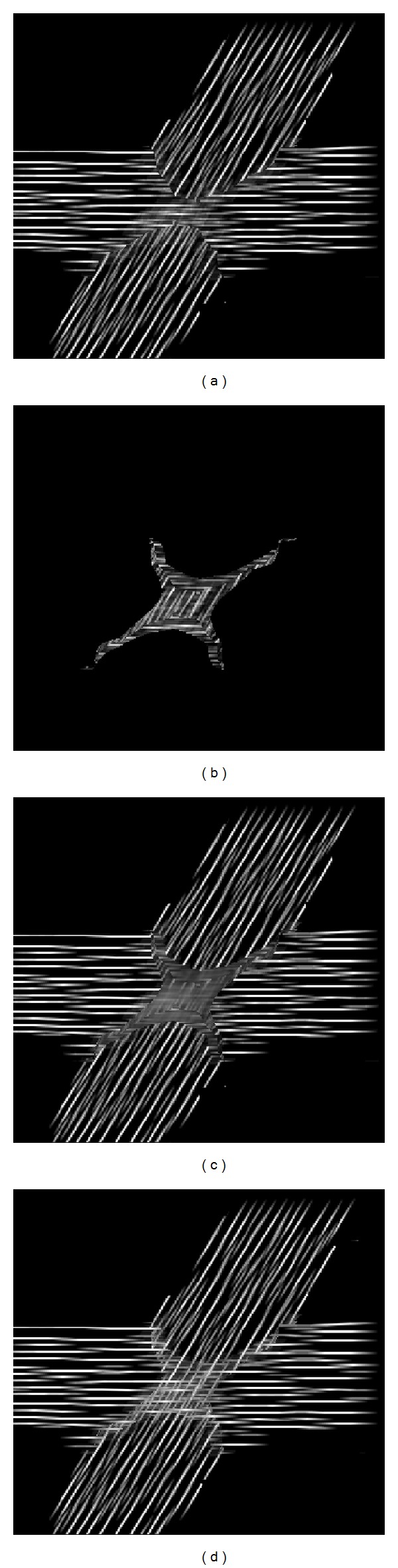
LIC results from HyperLIC and multiple-kernel LIC: first (a) and second (c) pass of HyperLIC, LIC result using the second direction only (b), and result from multiple-kernel LIC (d).

The LIC algorithm proposed by Cabral applies a one-dimensional filter kernel with a single flow direction per pixel. In order to take into account more than a single direction the standard LIC method must be adapted. Hotz et. al. [22] suggested generating one LIC result for every eigenvector field, integrating over the field's eigenvalues. All resulting images are overlaid to get a fabric-like texture. With HyperLIC Zeng and Pang proposed a multipass approach, where the LIC algorithm is applied to the principal eigenvector, using a noise texture as the input image to create an intermediate image [37]. In the second pass, the intermediate image is used as an input for LIC of the second eigenvector. Our experiments with an adapted HyperLIC algorithm yielded poor results, particularly in regions of crossing or branching fibers, which could not be clearly depicted (Figure 6(c)). In our method, a multi-directional LIC kernel is used. Firstly, integration is carried out along global FOD maxima over the input pattern. This yields the first smoothing result (Figure 6(a)). In a second step, integration is performed along the second local maximum of the candidate voxel, if there is a valid second FOD maximum. The resulting value (Figure 6(b)) is then combined with that from the first step. Combination of the two values can be performed by averaging. However, it was found that better contrast is achieved by setting the maximum of the two as the voxel's final LIC result (Figure 6(d)). Our multikernel approach can be implemented by generating a primary LIC image by application of a deterministic streamline tracking along the start voxel's global FOD maximum and generation of a second image with the second FOD maximum. The two images are subsequently combined by selecting the maximum of each pixel's two values. The start directions are found by analyzing the reconstructed FODs. During streamline tracking for every integration step the FOD maximum, that fits best with the direction from the previous step, is computed by a Newton-Raphson gradient ascent algorithm. For this, the spherical harmonics coefficients are linearly interpolated. Our multiple-kernel LIC uses a filter-kernel length of 15.

### 2.3. Visualization

When applied to a diffusion dataset, the LIC-based approach presented above, results in two highly resolved volume datasets: the gray-scale LIC volume and the direction volume of averaged anisotropy directions. For visualization of volume data, several approaches could be implemented. In the first method, data is projected into an image plane, for example, by volume rendering. In order to generate visualizations with sufficient contrast, the voxel's color and opacity have to be determined by suitable data characteristics. Examples include barycentric opacity mapping [36], threads and halos representations [38], and fiber tract coherence [39]. The second approach involves the generation of slice images, where slices orthogonal to the volume's main axes, oblique slices, or even curved slices are defined. Curved slices may be freely defined by the user or by anatomic structures, such as fiber pathways [40].

#### 2.3.1. Volume Rendering

Since the LIC volume produced by our method depicts fiber structures with good contrast, direct volume rendering by texture mapping is possible. For this purpose the LIC volume, which can be resampled to a lower resolution (to reduce the computational load) or clipped to a volume of interest, is loaded to the texture memory of the computer's GPU and rendered by texture mapping. The user can then interactively change the voxel transparency and color in order to highlight structures of interest.

#### 2.3.2. Directional Color-Coding

**Figure 7:**
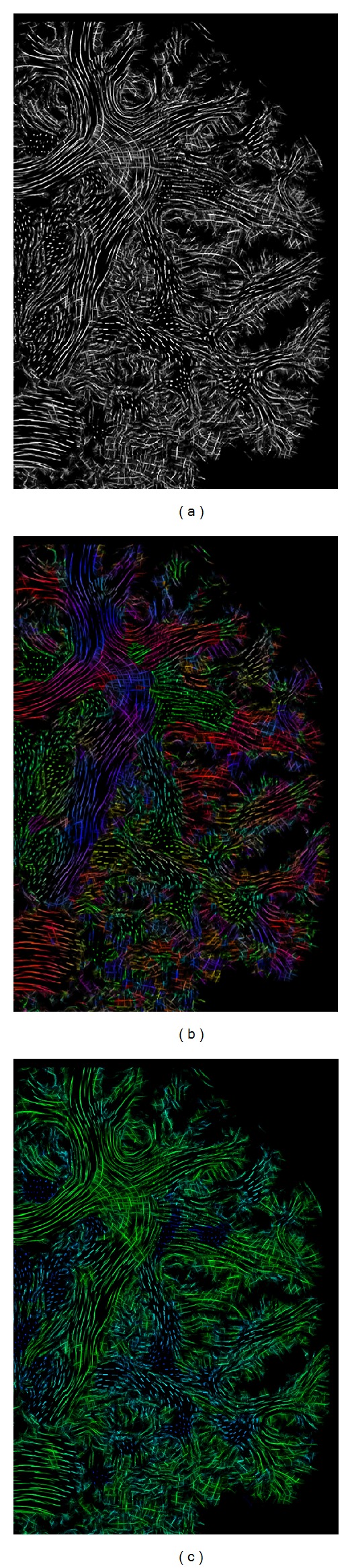
From left to right: section of a transaxial LIC slice image with gray scaling, and directional encoding with RGB and with HSB color model.

The easiest way to visualize LIC results is to present them as gray scale slice images depicting the structure of the underlying diffusion field (Figure 7(a)). However, these gray scale texture images do not exactly convey the direction of anisotropic diffusion in a pixel. In DTI, directional encoding is usually provided by color-coded FA maps, which combine the voxel's fractional anisotropy with the principal eigenvector direction of the diffusion tensor. Pajevic and Pierpaoli [1] proposed to assign the components of the eigenvector (*x*, *y*, *z*) to color channels red, green, and blue. Using this color model in LIC images results in blocked fiber continuity perception due to abrupt color changes (Figure 7(b)). Since the in-plane fiber direction is visualized by the structure of the LIC texture, no encoding of left-to-right or up-to-down directions is necessary. As a more appropriate alternative we use a color-coding scheme based on the hue-saturation-brightness (HSB) color model to encode different diffusion properties. HSB is a color representation with a cone-shaped color space. The hue channel is represented by a rotation angle with the main axis of the cone as the rotation axis, spanning from red (0/360) through green (120) to blue (240). The saturation channel is encoded as the fractional distance from the cone's center to its surface and defines the intensity of a color in a value range from 0 to 100. Brightness is measured from the cone's base (0 = black) to its tip (100 = white). In our color-coding scheme the LIC value is scaled from 0 to 100 and it defines the brightness channel. The direction is encoded as follows: when tracking along a streamline we compute the resulting direction vector ${\vec{v}}_{RN}$ as the sum of all the vectors $\vec{v}$ used for LIC tracking. The angle *γ* between the vector ${\vec{v}}_{RN}$ and the normal vector $\vec{r}$, orthogonal to the image slice, determines the hue value. |*γ*| is mapped from the [90,0] interval to the [120,240] color range. As a result, all streamlines within the slice are green in color and all streamlines orthogonal to the slice are colored in blue, thus avoiding continuity breaks (Figure 7(c)). We only use these two parameters and always set the saturation to 100.

#### 2.3.3. Slicing

**Figure 8:**
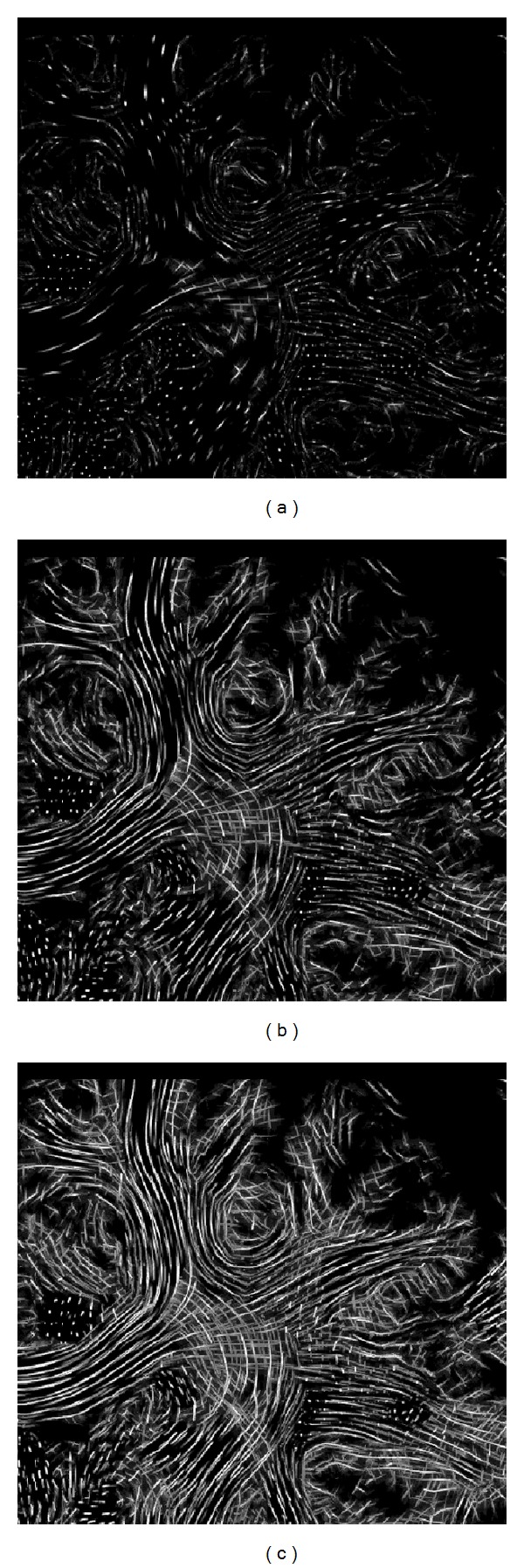
Combination of adjacent slices (zoomed details of transaxial slice image): single slice (a), 10 slices (b), and 20 slices (c) of the superresolution LIC dataset.

As explained in the previous section, it is useful to apply color-coding to slice images to encode anisotropy direction. When using slice thicknesses in the order of the resolution of the LIC volume, for example, <0.2 mm within one slice, the amount of information is low (Figure 8(a)). Therefore it is often useful to combine adjacent slices (Figures 8(b) and 8(c)). This is carried out by computing the average of the LIC values of adjacent pixels or, with even better contrast, by selecting the maximum value and then color-coding the pixel, as explained.

#### 2.3.4. Fusion with Anatomical Volume

**Figure 9:**
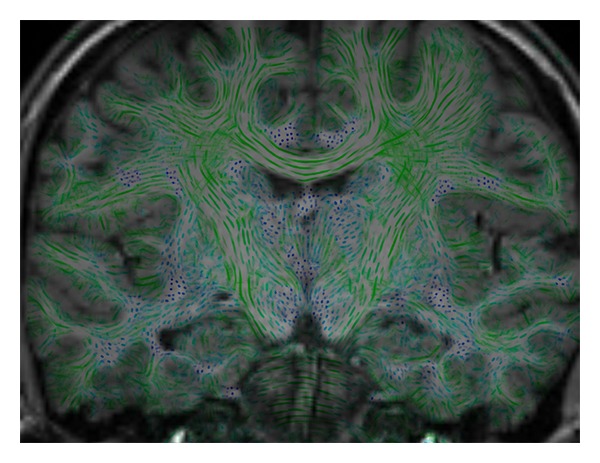
Fused directionally encoded LIC map and T1 slice image.

Further information can be gained by combining color-coded LIC slice images with slices from anatomical datasets, for example, T1 or T2 volumes (Figure 9). Usually, in a clinical setting a T1 volume is acquired together with a diffusion dataset. Clinicians can use the T1 data as an anatomical reference. Overlaying T1 slices with color-coded LIC slices implies that the two datasets have been registered and adjusted in spatial resolution. For fusion of the two slices we adapt the aforementioned color-coding scheme. In order to preserve T1 contrast, we rescale the T1 intensities from their original range from 0 to 4095 to the [0,100] brightness interval of the HSB color model. This is done by analysis of the gray scale histogram to determine the range of gray values covering 99% of the voxels and linearly mapping that range to the [0,100] interval. The voxel's LIC value is mapped to the saturation channel of the HSB model. The hue channel is used for directional encoding as explained above. As demonstrated by Figure 9, this coloring scheme allows T1 slices to be overlaid with fiber structure information without sacrificing T1 contrast. Instead of an automated scaling of the T1 intensities an interactive windowing procedure on a visualization console may be provided, thus enabling manual selection of window level and width.

### 2.4. Data Acquisition and Preparation

#### 2.4.1. Synthetic Data

Synthetic diffusion datasets were generated using a partial volume model similar to the one described in [41]. We chose a gradient *b*-value of 2000 s/mm^2^, a diffusivity of 0.0015 mm^2^/s, and a baseline signal *S*_0_ of 100. Furthermore, we used a free volume fraction of 0.35 in voxels completely occupied by fiber segments. We generated datasets containing multimodal voxels with fibers of 5 mm thickness crossing at different angles.

#### 2.4.2. *In Vivo* Data

For* in vivo* studies we used two different datasets. Diffusion data from the brain of a healthy 24-year-old human male was acquired on a 3-T Trio MR Scanner (Siemens Healthcare, Erlangen) using a spin-echo echo-planar diffusion-weighted sequence (TR = 8000 ms, TE = 105 ms) with 64 diffusion-encoding gradients, a *b*-value of 2000 s/mm^2^, and an isotropic spatial resolution of 2 mm. A data matrix of 108 × 108 was obtained measuring 56 slices. A second dataset from a clinical study of a 6-year-old child with a brain tumor in the left central region was acquired on a 1.5 T Vision Scanner using a spin-echo echo-planar diffusion-weighted sequence (TR = 11500 ms, TE = 122 ms) with 60 diffusion-encoding gradients, a *b*-value of 3000 s/mm^2^, and an isotropic spatial resolution of 2.5 mm. A data matrix of 96 × 96 was obtained measuring 60 slices. The child suffered from focal seizures due to this tumor and was scanned as part of a presurgical assessment. The MRI data were acquired as part of an ongoing study on the impact of early brain lesions. This study with its consent procedure was approved by the Ethics Committee of the Medical Faculty of the Eberhard Karls University of Tübingen. The parents gave informed written consent.

#### 2.4.3. Data Preparation

For the* in vivo* datasets we used a mutual information-based retrospective motion correction scheme in order to remove motion that occurred during the scan. For the simulated and* in vivo* datasets we computed the diffusion tensor for each voxel and additionally reconstructed the FOD with the constrained spherical deconvolution algorithm using a maximum spherical harmonic order *l*_max⁡_ = 8. We determined one or two main directions for each voxel by detecting the FOD's local maxima. To determine the local maxima of the FOD we reconstructed the FOD using 606 reconstruction directions, distributed over a hemisphere. A local maximum of the FOD is valid if (i) it reaches at least 50% of the global maximum, (ii) its direction does not deviate less than an angle of 30° from all previously found peaks, and (iii) all neighboring values on the FOD surface are smaller. The processing and visualization were performed on a Linux (Ubuntu 11.04) workstation with the following specifications: Intel Core i7-3630QM (8MB cache, 2 GHz × 8 cores with hyperthreading), 8 GB RAM (DDR3, 1333 MHz), and NVIDIA GeForce GT425 (with 1 GByte memory) graphics card. All processing steps were performed using the modular software platform OpenPDT developed by our group.

## 3. Results

### 3.1. Simulation Study

**Figure 10:**
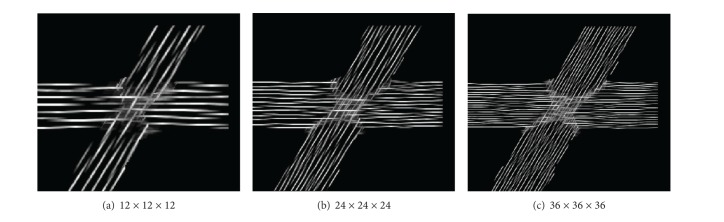
Influence of grid resolution on the LIC result using a resolution factor of (a) 1/12, (b) 1/24, and (c) 1/36.

On the basis of simulated datasets, generated as described above, we studied the robustness of the method against data characteristics, such as signal-to-noise ratio (SNR) and fiber pathways crossing angles. Moreover, we analyzed the effect of choosing different LIC grid resolutions and alternatives to FOD usage. Firstly, we generated two 5 mm thick fiber pathways crossing at an angle of 60 degrees. The diffusion dataset had an isotropic spatial resolution with a voxel length of 2 mm. This dataset was processed by LIC with grids of different resolutions: 1/12, 1/24, and 1/36 of the original voxel length. We used multicylindrical glyph samples with a length of 12 subvoxels and a width of 2.  Figure 10 shows the LIC results generated from the three resolutions. It is evident that with a finer superresolution grid we can place more cylinders and get a finer LIC texture, however, at the cost of longer computation times and greater memory consumption. From our experiments, a cylinder length of 12 subvoxels on a superresolution grid with a resolution factor of 1/24 was found to be a good tradeoff.

In a second experiment we used different glyphs to generate the LIC input:

1. normalized orientation distribution function (ODF),
2. sharpened ODF regularized by the ASSR approach described in [28],
3. FOD,
4. multiple cylinders derived from FOD maxima.

**Figure 11:**
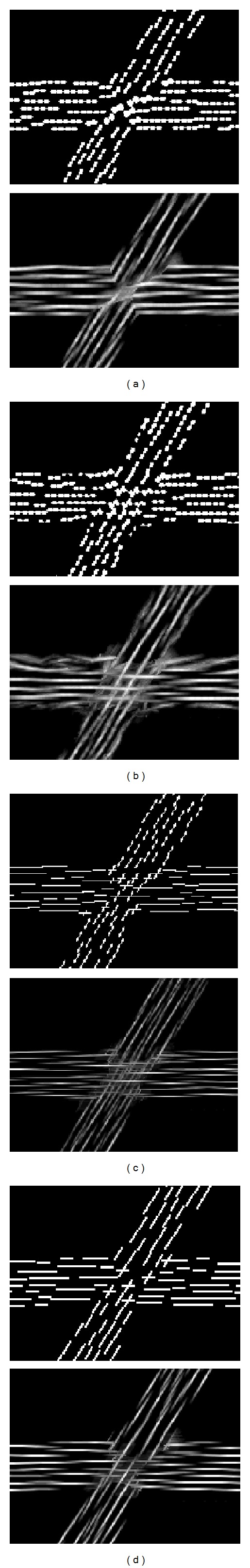
Results from the use of ODF (a), ASSR-sharpened ODF (b), FOD (c), and multiple cylinders (d). Input patterns (top) and their LIC results (bottom).

In Figure 11 the LIC results from these four glyphs, produced with a sample length of 12 subvoxels, are presented. By ODF sharpening the crossing region can be visualized more reliably. Even better results may be produced by use of the FOD or multiple cylinders.

**Figure 12:**
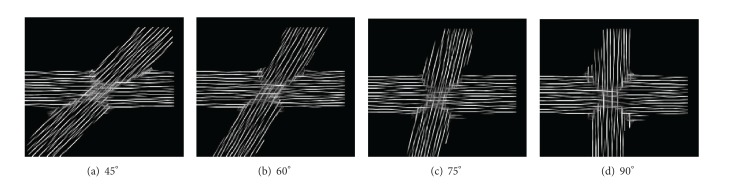
LIC results of two fibers crossing at different angles.

Thirdly, we simulated two 5 mm thick fiber pathways crossing at angles of 45, 60, 75, and 90 degrees. The datasets were processed with multiple-cylinder glyphs with a length of 12 subvoxels and a grid resolution factor of 1/24.  Figure 12 shows that for these crossing angles the method is capable of correctly visualizing the crossing.

**Figure 13:**
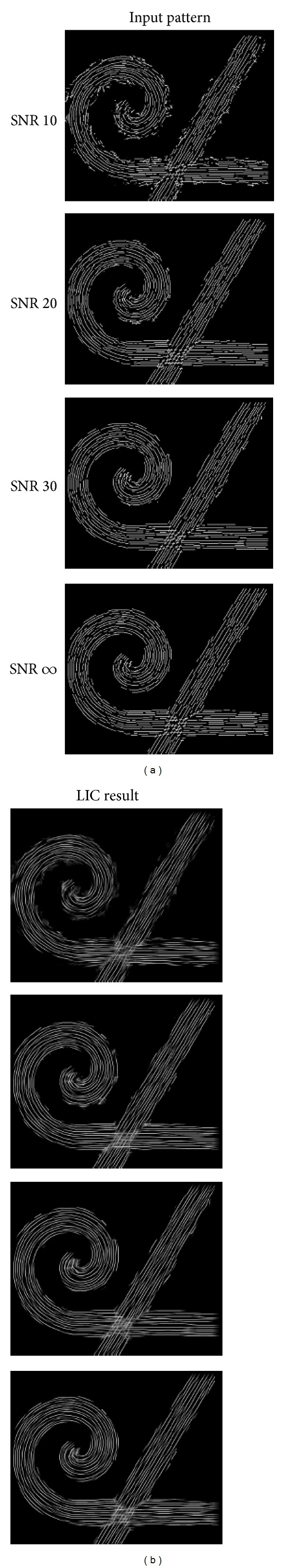
Results from the simulation study demonstrating the influence of SNR on multicylindrical glyph sample (a) and LIC result (b).

In our last simulation experiment we created datasets containing two fiber pathways, one with a spiral part, crossing at an angle of 60 degrees, and added different amounts of noise resulting in a signal-to-noise ratio (SNR) between 10 and *∞*.  Figure 13(a) shows the multicylindrical glyph pattern produced with a grid resolution factor of 1/20, a cylinder length of 12 subvoxels, and a width of 2. The LIC results (Figure 13(a)) show that the method is relatively robust against the influence of noise. This is due to the fact that the LIC algorithm uses a smoothing approach to engrave the flow field's structure onto the input texture, thus suppressing noise.

### 3.2. *In Vivo* Study

**Figure 14:**
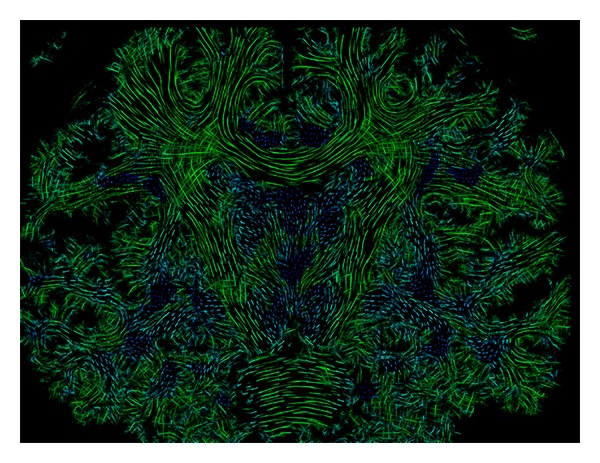
Coronal view of a color-coded slice of healthy volunteer.

We applied the proposed approaches of anisotropic multicylindrical glyph sample generation, multikernel LIC, and color-coding to both of the acquired* in vivo* diffusion datasets described above. In both cases we used a resolution factor of 1/24 and a cylinder length of 12 subvoxels.  Figure 14 shows a color-coded coronal slice from the healthy volunteer dataset. The slice thickness used was 2.0 mm. Major fiber pathways, such as pyramidal tract and corpus callosum, were depicted with good contrast, including the crossing of callosal projections with pyramidal fibers. The blue structures represent fibers running orthogonal to the slice plane, such as the cingulum.

**Figure 15:**
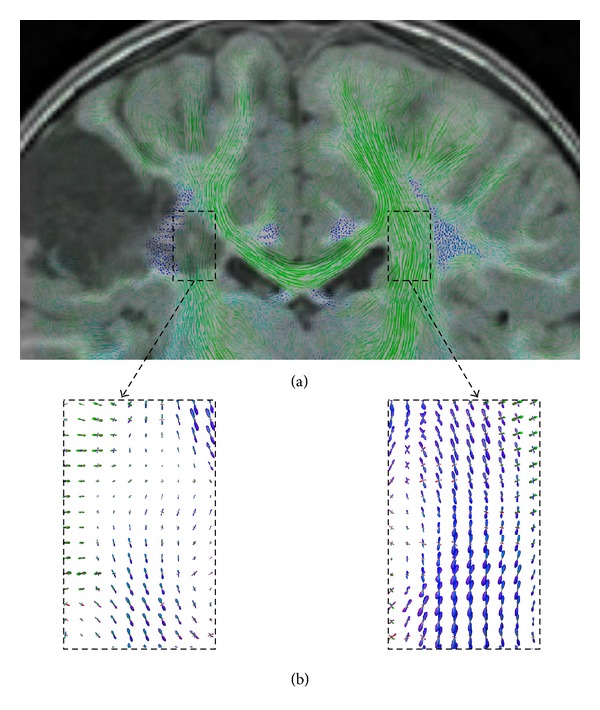
*In vivo* dataset with tumorous tissue infiltrating the right branch of the pyramidal tract: fused directionally encoded LIC map and T1 slice (a) and FOD glyphs from both pyramidal tracts (b).

The LIC result from processing the tumor patient dataset was fused with the T1 data by application of our color-coding scheme (Figure 15(a)). The figure shows that the T1 contrast could be preserved. The left box indicates a region in which tumorous tissue evidently infiltrated the right branch of the pyramidal tract, without having totally destroyed anisotropic behavior. The glyph representation (Figure 15(b)) of the affected region demonstrates a loss of anisotropy, indicated by considerably smaller FODs as compared to a corresponding region of healthy tissue. This information was of great value for surgeons during operation planning. In addition, the intraoperative electric stimulation confirmed that the fibers within the solid part of this dysembryoplastic neuroepithelial tumor (WHO grade 1) were in fact pyramidal tract fibers. Consequently, a subtotal resection was performed, with only a small part of the tumor remaining. The patient had no postoperative sensorimotor deficit.

## 4. Discussion and Conclusions

We have presented an approach for fiber visualization by applying an LIC algorithm. To generate meaningful LIC maps which reliably represent fiber structures, novel pre- and postprocessing methods were proposed. Firstly, the tensor model was dismissed and instead an FOD representation of the local diffusion profile was used, due to its capability of describing multiple anisotropy directions. We used FOD or alternatively multicylindrical glyph samples, rasterized by a superresolution grid, and thus were able to impress anisotropy characteristics into the LIC input pattern and substantially improve the contrast of LIC maps. Secondly, we proposed a sample placement strategy, which uses a uniform random technique to distribute seeds over the data volume and sets samples along integration lines that were produced by deterministic tracking over very short distances. This allowed the continuity of fiber lines in LIC maps to be enhanced. Thirdly, a color model and a color-encoding scheme for the directional encoding of LIC maps and their fusion with slices from anatomic datasets were presented. Users of this technology may, for example, view T1 slices with superimposed LIC maps and thus relate macroscopic anatomy to superresolved fiber structure. In contrast to other methods which produce white matter images with high spatial resolution, we did not consider global connectivity features but focused instead on local diffusion characteristics. Previous track density imaging (TDI) studies have utilized the long-range information from fiber tracks to generate highly resolved TDI maps [42]. TDI applies probabilistic tracking over long distances to create fibers. Since fiber tracking algorithms with multiple parameters essentially perform interpretations of the diffusion data, they are prone to errors, and error propagation along tracks may lead to misinterpretations of data [43]. In TDI, this problem is addressed by generating a multitude of streamlines, typically in the order of millions, assuming that erroneous tracks are smoothed out by the multitude of correct fiber lines. In our LIC approach we do not perform global interpretations of the diffusion data. Rather, data is processed by algorithms, which operate on a local level. Indeed, tracking is performed for LIC kernel generation and for continuous sample placement, but only over very small distances in the order of the voxel size of the original diffusion dataset. Errors may occur, for example, during FOD maximum detection, but since tracking stops after a few iterations, error propagation is not a significant problem in this LIC approach. The approach does not perform global interpretations, such as streamlines, and therefore can not be used to analyze and verify the connectivity between brain regions, for example, by tracking streamlines from a seed to a goal regions. Hence it cannot substitute global methods but may help to study regional fiber architectures. As explained in the introduction there have been a few previous approaches to apply LIC methods to DW-MRI datasets. Particularly, by our novel techniques of (1) generating MR diffusion specific anisotropic input patterns and (2) using multiple LIC kernels, we could substantially improve the contrast and expressiveness of resulting LIC maps, thus allowing fiber pathways to be discerned even in complex regions of branching and crossing fibers.

Since we apply LIC to a highly resolved 3D sample pattern with a grid resolution that surpasses the spatial resolution of the original diffusion dataset by an order of magnitude, the computational workload of the LIC step of our method is quite high. With a typical whole brain diffusion dataset consisting of more than 50 slices and a spatial resolution of approximately 2.0 mm, resulting processing times are of the magnitude of several hours on standard desktop computers. It was necessary to speed up the LIC processing and thus our method was implemented with an adapted FastLIC algorithm [44], making the process up to 5 times faster. Another solution would be the transfer of LIC processing to graphics processing units (GPUs) and usage of their high memory bandwidth and high degree of parallelism. Since for each voxel of the superresolution grid the same processing steps have to be performed, it is easy to parallelize the algorithm and exploit a GPU's parallel processing architecture. With GPU implementation of the algorithm we could speed up the process by a factor of approximately 90. Further speeding up is possible by usages of multiple GPUs.

The visualization strategy of presenting LIC maps has not been widely used in DW-MRI. Therefore, we cannot rely on previous practical experience with this kind of methodology and we are aware of the fact that the method may generate visual stimuli that lead to erroneous interpretations of the diffusion data. However, it is hoped that its practicality by a thorough evaluation of the method can be demonstrated by further clinical studies. We think that it is worthwhile to further investigate the application and adaptation of texture-based vector field visualization methods, not only LIC, but also other methods, such as spot noise [45] and texture splats [46].
